# Effects of Different Growth Regulators on the Rooting of *Catalpa bignonioides* Softwood Cuttings

**DOI:** 10.3390/life12081231

**Published:** 2022-08-15

**Authors:** Jin’e Quan, Ruoyi Ni, Yange Wang, Jiajia Sun, Mingyue Ma, Huitao Bi

**Affiliations:** College of Forest, Henan Agricultural University, Zhengzhou 450002, China

**Keywords:** *Catalpa bignonioides*, softwood cutting, growth hormone, rooting index

## Abstract

(1) Background: To further improve the rapid reproduction and large-scale application of *Catalpa bignonioides*. (2) Methods: With young softwood cuttings from a 3-year-old *C. bignonioides* mother plant used as materials, the effects of indole-3-acetic acid(IAA), indolebutyric acid(IBA) and rhizogenic powder-1(ABT-1) growth regulators at different concentrations on cutting indexes and the dynamic changes in endogenous hormone contents during the rooting of the *C. bignonioides* cuttings were studied. (3) Results: The rooting of *C. bignonioides* cuttings could be divided into five stages. There were three types of rooting of adventitious roots. IBA treatment resulted in a high rooting rate and beneficial root morphology. The morphological indexes of the cutting roots after treatment with 1000 mg·L^−1^ IBA had the best overall quality, which was significantly higher than that of the roots in the control (CK) group (*p* < 0.05). Although the average longest root length (20.51 cm) under ABT-1 was the longest, its overall average rooting rate was slightly lower than that under IBA. The rooting effect under IAA was generally lower than that under IBA and ABT-1. The endogenous hormone content of the cuttings was found to be closely related to rooting; the IAA and zeatin nucleoside (ZR) content was high, and the ratios of IAA/ABA and IAA/ZR were high. The contents of gibberellin_3_ (GA_3_) and abscisic acid (ABA) were low, which had a promoting effect on the rooting of the cuttings. (5) Conclusions: All three kinds of auxin can promote rooting and, of the three treatment groups, the rooting effect of cuttings in the IBA treatment group was the strongest, with 1000 mg·L^−1^ being the optimum concentration.

## 1. Introduction

*Catalpa bignonioides*, also known as cigar tree, American catalpa and Indian bean tree, is a deciduous tree species of the family Bignoniaceae and is native to North America. *C. bignonioides* is grown mainly in parts of Canada and the central and southern United States. In recent years, *C. bignonioides* has also been introduced in the northern region of China to central Xinjiang, Heilongjiang, Jilin, Liaoning, and south to Yunnan and other provinces [1]. Owing to its strong vigor, large leaves, and floral fragrance, as well as its ability to produce dense shade, *C. bignonioides* is mainly used as garden ornamental or street tree. Its flowers are white and bell-shaped, with yellow or lavender spots. The pods are similar to long beans and hang throughout the tree canopy, which is very attractive. There are different leaf colors, including green, yellow, and purple, as well as multicolored leaves. Attractive features of these trees are visible in three seasons, namely, its leaves in the spring, its flowers in the summer, and its fruits in autumn. The leaves are large and rough and have the ability to strongly absorb particles and heavy metals, such as lead and cadmium. Owing to its broad canopy, *C. bignonioides* is often used as a wind barrier [2]. Wei Zuoping et al. [3] reported that *C. bignonioides* is an important primary landscaping tree species with optimal landscape effects. Bi Huitao et al. [4] treated softwood cuttings of *C. bignonioides* with different concentrations of IBA, and its rooting rate increased by 84% compared with that of the cuttings in the CK group. In recent years, although research on *C. bignonioides* has mainly focused on trees of the same family and genus, relatively few studies have focused on seedling propagation [4]. Chen Suchuan et al. [5] and Liang Youwang et al. [6] conducted preliminary research on the sowing of *Catalpa* species and the rooting of softwood cuttings, and Zhao Xiyang et al. [7] and Peng Chan et al. [8] explored the seed germination characteristics of *Catalpa eucalyptus* and the effects of exogenous hormones on the growth of *Eucalyptus* seedlings. Cui Lingjun et al. [9] carried out subjected *Catalpa bungei* seeds to different hormone treatments and concluded that IBA promotes seed germination. However, the speed of propagation via seed is slow due to the dormancy characteristics of these species [3]. In addition, limited domestic seed resources prevent rapid and large-scale promotion and application. The cutting propagation technique has advantages that include the stable maternal inheritance of excellent traits, a short growth cycle, ease of operation, and rapid prototyping; thus, propagation by cuttings has been considered to be the most cost-effective reproduction method in recent years and can hasten and promote the propagation of large numbers of seedlings [10]. Therefore, the cutting propagation technique is beneficial to accelerate the promotion and application of *C. bignonioides* reproduction.

At present, there has been no research on *C. bignonioides* cutting propagation techniques in China and other countries. The low rooting rate of cuttings has been a major bottleneck in forestry development in China. To overcome this limitation, many researchers have carried out related research on the effects of growth regulators on various species, such as *Chionanthus virginicus, Lonicera korolkowi, Quercus mongolica, Mytilaria laosensis*, and other tree species. According to the research results, the rooting rate of cuttings treated with exogenous growth regulators was found to be significantly higher than that of untreated cuttings [11,12,13,14]. The selection and application of auxin is also a key factor for improving the rooting rate of cuttings. In addition, treatment with growth regulators whose concentrations are too high or too low is not conducive to the rooting of cuttings [15] because low or high concentrations of exogenous growth regulators can disrupt the balance of endogenous hormone contents within the cutting itself, resulting in an inability to promote adventitious root production [16]. Wang Qing et al. used *Chukrasia tabularis* shoots cultured in MS-containing medium as the research object and found that adding IBA and ABT-1 at a concentration of 500 mg·L^−1^ induced rooting after 10 days. Furthermore, IBA processing outperforms ABT-1 processing, with a rooting rate over 85% [17]. By using three kinds of growth regulators (naphthaleneacetic acid (NAA), IBA, and IAA) at different concentrations and treatment times, Zhai Yafang et al. conducted a cutting propagation test on *Lonicera tartarii* cuttings. According to the test results, the rooting rate under IBA 500 mg·L^−1^ was the best at 86% [18]. Wang Xiaoling et al. soaked tetraploid *Robinia pseudoacacia* softwood in different concentration gradients (500 mg·L^−1^, 1000 mg·L^−1^, 1500 mg·L^−1^) of different hormones (IBA, NAA, and IAA) for 6 h. The best treatment group was IBA at a concentration of 1000 mg·L^−1^ with a rooting rate of 80.4% [19]. Zhang Enliang treated *Catalpa* softwood with IBA at a concentration of 2000 mg·L^−1^, with a rooting rate of 85.6% [20]. Ma Lingling et al. evaluated the cutting rooting ability of five species of *Catalpa* softwood. After soaking in IBA with a concentration of 3000 mg·L^−1^ for 1 min, the rooting rate of *Catalpa* was as high as 94.5% [21]. Wang Gaiping used NAA, IAA, and ABT-1 at a concentration of 1000 mg·L^−1^ to analyze the rooting development and root characteristics of the cuttings of *Catalpa* softwood. Among them, the ABT-1 treatment group with a concentration of 1000 mg·L^−1^ was the best with a rooting rate of 92% [22]. Zhang Mei et al. used different concentrations of ABT-1, NAA, and IAA to treat 1-year-old cuttings of *Brassica chinensis*. Among them, the rooting rate of ABT-1 with a concentration of 500 mg·L^−1^ was the highest, followed by the IAA treatment group [23]. In order to determine a suitable cutting plan for cold-resistant plum root stocks, Wang Xuesong et al. soaked the rooting powder in NAA, IAA, IBA, and ABT-1 at concentrations of 1, 3, and 5 g·L^−1^ for 40 min. The best treatment group was ABT-1 at a concentration of 3 g·L^−1^ [24]. Liang Xiaochun et al. used different concentrations of the exogenous hormones IAA and IBA to treat Huangguohougui softwood and found that the IAA treatment group with a concentration of 250 mg·L^−1^ was the best, with a rooting rate of 66.7% [25]. Li Huimin et al. studied the effect of different concentrations of plant growth regulators (IBA, IAA, and NAA) on the rooting survival rate of cuttings of original varieties of perfume rose. Among them, the survival rate of cuttings treated with IAA solution with a concentration of 500 mg·L^−1^ was the highest [26]. Wu Kaiyun et al. used various concentrations of plant growth regulators (IBA, ABT-1, NAA, shuang ji er-GGR6, and IAA) to soak hardwood cuttings from the persimmon rootstock Yalin 6 and then inserted them into a sterile matrix. The highest rooting rate was found for the IAA treatment group with a concentration of 500 mg·L^−1^, followed by the ABT-1 treatment group [27]. The best growth regulators widely used to rapidly increase the rooting rate of cuttings are IBA, ABT-1, and IAA, which are suitable at concentrations ranging from 500 mg·L^−1^ to 2000 mg·L^−1^.

According to a large number of studies in recent years, the changes in the contents of endogenous hormones during the rooting process of cuttings are more important for regulating the occurrence of adventitious roots than their contents in a specific developmental period. In addition, treatment with growth regulators whose concentrations are too high or too low is not conducive to the rooting of cuttings [28,29]. In a study by Zhang Xiaoping et al. [30] on *Liriodendron chinense ×* L. *tulipifera* and a study by Li Chaochan et al. [31] on *Rhododendron stamineum*, IAA was found to promote the formation of adventitious roots and ABA had a certain inhibitory effect on root formation. Exogenous hormone treatment can promote the synthesis of endogenous IAA and inhibit the synthesis and transport of endogenous ABA. In research on *Rhododendron scabrifolium* [32] the increase in the GA_3_ content had a positive correlation with the induction of cutting calli and the occurrence of adventitious roots. According to an experiment on *Swida wilsoniana* by Li Yongxin et al. [33] a high concentration of ZR is beneficial to the growth and development of root primordia, while a low concentration of ZR is beneficial to the formation of root primordia. In research by Dong Shengjun et al. [34] on *Armeniaca sibirica* and by Shi Fenghou et al. [35] on *Tilia miqueliana*, plant growth regulators could affect the changes in endogenous hormones in cuttings and promote cutting rooting.

With the use of young softwood cuttings of 3-year-old *C. bignonioides* mother plants as materials, this experiment involved applying three different growth regulators, namely, IAA, ABT-1, and IBA, at concentrations of 500 mg·L^−1^, 1000 mg·L^−1^, and 1500 mg·L^−1^ on the materials to study their impact on the rooting index of the cuttings. The cuttings displaying optimal rooting across the treatment groups were screened, and the dynamic changes in the contents of four main endogenous hormones IAA, ABA, GA_3_, and ZR and their ratios during the rooting process were measured. This study identified the best growth regulator to deal with the rooting rate of cuttings and the survival rate of *C. bignonioides* softwood for the first time and provided a theoretical basis for future studies. From the perspective of physiology and biochemistry, the rooting mechanism was explained and can be used for the establishment of a rapid propagation technology system of *C. bignonioides* seedling cuttings and the rapid large-scale production of seedlings with excellent genetic traits. Moreover, technical support and theoretical guidance on the industrialization development of *C. bignonioides* were provided.

## 2. Materials and Methods

### 2.1. Test Materials, Cutting Treatments, and Sample Collection

In this research, the materials were softwood cuttings of *C. bignonioides* collected in mid-July from a 3-year-old mother plant at an experimental station in Ruzhou city, Henan Province. The mother plants were all similar in terms of their height. The cuttings were collected from vigorously growing, young branches and were 0.6–0.8 cm in diameter, after which they were all pruned to a length of 12–15 cm. The upper end of each cutting was cut flat, and the base incision was beveled at 45°.

Three growth regulators, ABT-1(Beijing EbTY Biotechnology Co., Ltd., Beijing, China), IAA, and IBA (Beijing Solaibao Technology Co., Ltd., Beijing, China) were applied separately. IAA, IBA, and ABT-1 at 0.5 g, 1 g, and 1.5 g, respectively, were dissolved with 100% alcohol and then diluted with 1 L of water to a concentration of 500, 1000, and 1500 mg·L^−1^ to soaking 2–3 cm of the cutting’s base for 1 h. There were 9 treatments, each of which was replicated 3 times. A total of 80 cuttings constituted a bundle (one replication). Cuttings soaked in water for 1 h were used as CKs, as shown in Table 1.

For each bundle of 80 cuttings, the base was first soaked in 50% carbendazol wettable powder (Jiangsu Lanfeng Bio-Chemical Co., Ltd., Nanjing, China) diluted 800 times with water for 1 min. To accurately observe the morphological changes in the base of the cuttings, three cuttings were randomly selected from each treatment every other day for observations, and the rooting status of the cuttings was recorded. Sampling was performed on days 0, 7, 15, 30, and 50 after treatment. Three cuttings were randomly selected for each treatment. First, the cuttings were rinsed with water and dried. Then, the cortex within 2 cm of the cutting was immediately removed. After being cut into pieces, they were wrapped in aluminum foil and put into liquid nitrogen. Finally, the cuttings were stored in a −80 °C ultra-low-temperature freezer in the laboratory for the subsequent determination of endogenous hormone contents.

### 2.2. Cuttings and Post-Management Processes

In this study, a softwood cutting system used for breeding was adopted. A seedbed with a length of 5 m, a width of 4 m, and a depth of 0.5 m surrounded by bricks was used. The matrix in the seedbed consisted of pure fine river sand:vermiculite:perlite = 3:1:1. An automatic spraying system was installed 1.5 m above the seedbed, and a shading net was placed 10 cm above the automatic spraying system. One week before the cuttings were transplanted, 50% carbendazol wettable powder diluted 800 times with water was evenly sprayed around the seedbed and the substrate. At the same time, the substrate was tumbled. Before the cuttings were transplanted, the seedbed substrate was watered to loosen the matrix particles. The cuttings were transplanted such that the density was 40 m^−2^ and their depth was 7–8 cm. After the cuttings were transplanted, water was sprayed every 30 min from 8:00 to 18:00 every day. To prevent excessive water spraying or high temperatures and water shortages in the summer, a moisture timer controller produced by staff at the Beijing Academy of Forestry was used to monitor the water spraying intervals and times (Chinese Academy of Forestry Sciences, Beijing, China). On sunny days from 10:00 to 16:00, a shade net was placed over the cuttings. The moisture of the cutting substrate was moderate. Moreover, the relative humidity at 1.5 m above the cuttings was maintained at approximately 70%.

### 2.3. Determination of Rooting Indicators

Beginning on day 5, three cuttings were randomly taken each time to observe the changes in cutting morphological indicators. On day 50, the rooting of all the cuttings in the seedbed was investigated. First, the counting method was used to quantify the callus production rate and rooting rate in each cutting group. Then, an Epson root analyzer (Epson Perfection 4990 Photo scanner, Epson, Nagano, Japan) manufactured in Nagano, Japan, was used to determine the root number, maximum root diameter, and maximum root length of each cutting group.

Rooting rate (%) = (number of rooted cuttings/total number of cuttings tested) × 100%(1)

Callus production rate (%) = (number of cuttings that produced calli/total number of cuttings) × 100%(2)

Root number = sum of root number/total number of roots(3)

Average maximum root diameter (mm) = sum of maximum root diameter/total roots(4)

Average maximum root length (cm) = sum of maximum root length/total number of roots(5)

### 2.4. Determination of Physiological and Biochemical Indicators

For the determination of physiological indicators of the phloem of *C. bignonioides* cuttings, the method proposed by He Chongdan et al. [29] was used to extract endogenous hormones. Enzyme-linked immunosorbent assays (ELISAs) were used to determine the contents of rooting-related endogenous hormones, including IAA, GA_3_, ABA, and ZR, in units of nanograms per gram of fresh weight (FW). Each sample was replicated 3 times.

### 2.5. Data Processing

SPSS 24.0 software (IBM Corp., Armonk, NY, USA)was used for analysis of variance and correlation analysis. Origin 8 software(Originlab, Northampton, MA, USA) was used for mapping. Duncan’s method was used for multiple comparisons, and the mean ± SE was used to represent the test results.

## 3. Results and Analysis

### 3.1. Rooting of C. bignonioides Softwood Cuttings

**Figure 1:**
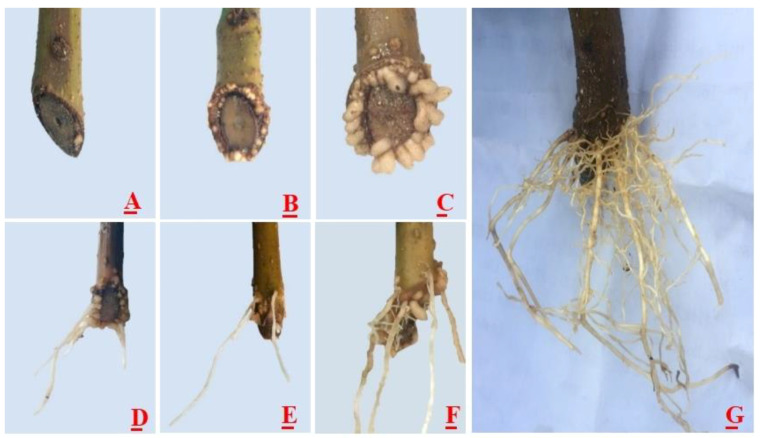
Development process of adventitious roots of *C. bignonioides* cuttings. (**A**) A small amount of callus was generated; (**B**) A large number of calli were generated; (**C**) Callus increase; (**D**) Adventitious roots protruding from the calli; (**E**) Adventitious roots protruding from the bark; (**F**) Calli and bark of the same cutting producing adventitious roots; (**G**) Calli and the bark of the same cutting producing adventitious roots that have elongated.

The morphological changes in the *C. bignonioides* cuttings are as follows. Beginning on day 10, some cutting bases produced a small amount of milky calli (Figure 1A). On day 15, the calli gradually increased (Figure 1B) and on day 20, the calli continued to gradually increase (Figure 1C). On day 30, some adventitious roots continued to extend from inside the calli (Figure 1D). A few adventitious roots had been generated from the bark of the cuttings (Figure 1E). Some adventitious roots arose from the interior and cortex of the calli of the same cutting (Figure 1F) and elongated. In addition, new roots had formed on these roots. Finally, a complex interlaced root system formed (Figure 1G). According to the phenotypic changes in the rooting of the *C. bignonioides* cuttings (Figure 1), the process could be divided into five stages, namely, the initiation stage, callus stage, root primordium induction stage, adventitious root generation period, and elongation stage. According to previous research, the different rooting types can be divided into bark rooting types, callus rooting types, and mixed rooting types [16,36]. Figure 1 shows that the rooting process of the *C. bignonioides* cuttings involves these three rooting types.

### 3.2. Comparison of Rooting Rate and Callus Production Rate in Response to Different Growth Regulators

#### 3.2.1. Influence of Growth Regulator on the Rooting Rate and Callus Production Rate of *C. bignonioides* Softwood Cuttings

Table 2 shows that the rooting rate and callus production rate of *C. bignonioides* cuttings under the three growth regulator treatments were higher than those under the CK treatment, with a consistent trend. The impact of the growth regulators on these two indicators was in the order of IBA > ABT-1 > IAA. Among the cuttings under the three growth regulators, the rooting rate under IBA was 62.44%, which was 7.81 times that under the CK and was significantly greater than that under the other growth regulators. Moreover, the rooting rate in response to ABT-1 was significantly greater than that under IAA. The callus production rate under IBA was 18.44%, which was 9.22 times that under the CK; this production was not significantly different from that under ABT-1 but was significantly greater than that under the other growth regulator treatments. The difference between the IAA and CK treatments was not significant. Overall, the rooting rate under the three growth regulators treatments was greater than the callus production rate, and the IBA treatment elicited the best response.

#### 3.2.2. Effects of Growth Regulator Concentration on the Rooting Rate and Callus Production Rate of *C. bignonioides* Softwood Cuttings

**Figure 2:**
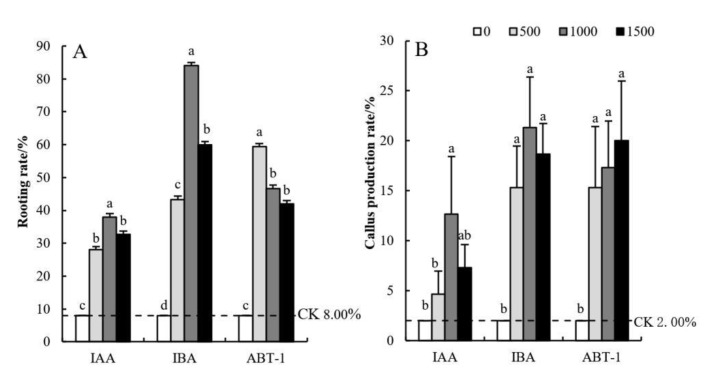
Effects of different growth regulator concentrations on the rooting rate and callus production of *C. bignonioides* softwood cuttings. (**A**) The rooting rate of cuttings treated with different growth regulators, (**B**) The callus production rates of cuttings treated with different growth regulators, IAA: Indole-3-acetic acid, IBA: Indolebutyric acid, ABT-1: Rhizogenic powder-1. a, b, c: Different superscripts show significant differences (*p* < 0.05) and the same letters indicate no difference (*p* > 0.05). Bars represent the standard error (n = 3).

The rooting rate of cuttings treated with different growth regulators is shown in Figure 2A. Overall, the rooting rate of cuttings in the IBA treatment group was the best (43–84%) and was significantly higher than that of the cuttings in the IAA and ABT-1 treatment groups. With an increase in IBA concentration, the rooting rate tended to first increase and then decrease. Among the different concentrations tested, the maximum rate was 84.00% in response to 1000 mg·L^−1^, which was 90.48% higher than that under the CK (the rooting rate of which was 8.00%). The second greatest rooting rate was detected in the ABT-1 treatment group. With the increase in ABT-1 concentration, the rooting rate also increased first and then decreased. The rooting rate of the cuttings was the best after treatment with 500 mg·L^−1^ ABT-1 compared with other concentrations, which was 86.52% higher than that of the CK. In general, the rooting rate of the IAA treatment group was the lowest (28–38%); IAA had no significant effect on the rooting rate.

As shown in Figure 2B, the callus production rates of the cuttings in the IBA and ABT-1 treatment groups were similar, and the difference between the different concentrations of hormones was not significant (15–22%); however, the production rates in response to both were significantly higher than that of the CK. Among the treatment groups, the callus production rate of the IBA treatment group first increased and then decreased with increasing concentration, which is consistent with the rooting rate trend shown in Figure 2A. The callus production rate of the cuttings in the ABT-1 treatment group showed a gradually increasing trend with increasing concentration, which is different from the rooting rate trend shown in Figure 2A. The cuttings in the IAA treatment group presented the lowest callus production rate (4–13%), which was lower overall than that in the IBA and ABT-1 treatment groups. In addition, the overall trend and significance between the different hormone concentrations were essentially consistent with those of the rooting rate, as shown in Figure 2A.

### 3.3. Comparison of Root Morphology in Response to Different Growth Regulator Treatments

#### 3.3.1. Effects of Growth Regulators on Root Morphology of *C. bignonioides* Softwood Cuttings

The root morphological changes of the *C. bignonioides* softwood cuttings are shown in Table 3. The three growth regulators significantly affected the number of roots produced, average maximum root length, and average maximum root diameter of the cuttings. In the IBA treatment, the number of roots produced reached 16.56, which was 2.37 times that in the CK. This was significantly greater than the numbers in the ABT-1 treatment (10.11 roots) and IAA treatment (8.33 roots) (*p* < 0.05). Second, ABT-1, IBA, and IAA all significantly promoted the average maximum root length of the *C. bignonioides* softwood cuttings. Among these treatments, the promoting effect of ABT-1 was the strongest (20.51 cm on average), which was significantly higher than that of CK (10.40 cm). Finally, the average maximum root diameter in response to the three growth regulators ranged from 1.27–1.61 mm, which equates to the roots being 41.67~58.82% thicker than those of the CK (0.7 mm on average). Therefore, overall, the hormone treatment of *C. bignonioides* softwood cuttings has a significant promoting effect on maximum root diameter. Among the treatments, the effect of IBA was the strongest and was significantly better than that of ABT-1.

#### 3.3.2. Effects of Growth Regulator Concentration on the Root Morphology of *C. bignonioides* Softwood Cuttings

**Figure 3:**
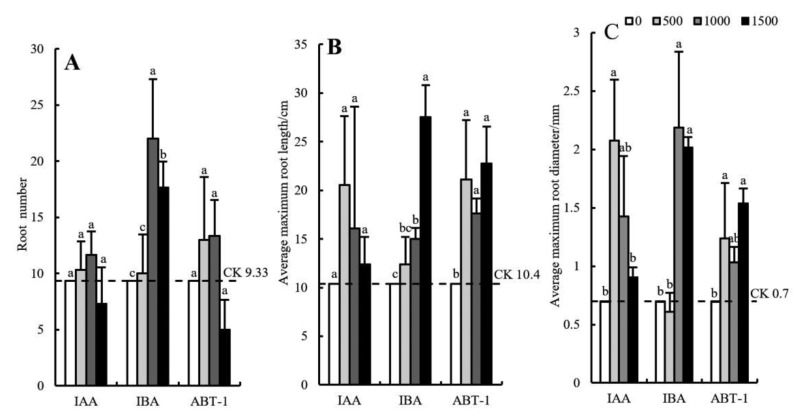
Effects of growth regulator concentrations on the root morphological indexes of *C. bignonioides* softwood cuttings. (**A**) The number of roots produced by the cuttings treated with different growth regulators, (**B**) The average maximum root length by the cuttings treated with different growth regulators, (**C**)The average maximum root diameterby the cuttings treated with different growth regulators, IAA: Indole-3-acetic acid; IBA: Indolebutyric acid; ABT-1: Rhizogenic powder-1. a, b, c: Different superscripts show significant differences (*p* < 0.05) and the same letters indicate no difference (*p* > 0.05). Bars represent the standard error (n = 3).

As shown in Figure 3A, the number of roots produced by the cuttings in the IBA treatment group was the highest. In addition, the number of roots in response to IBA at concentrations of 1000 mg·L^−1^ and 1500 mg·L^−1^ were significantly higher than those in response to ABT-1 and IAA. The number of roots first increased and then decreased slightly with increasing IBA concentration. The maximum value was 22.00 under the 1000 mg·L^−1^ IBA treatment, which was approximately 2.36 times that under the CK treatment. Therefore, IBA concentrations that are too high or too low are not conducive to the development of adventitious roots. The effects of the ABT-1 and IAA treatments on the number of roots produced were relatively weak (5~14 roots); there was no significant difference in number of roots produced by the cuttings between the concentrations of these hormones or between the CK. Therefore, the promoting effect of ABT-1 and IAA on the number of roots was not obvious. When the IAA and ABT-1 concentrations reached 1500 mg·L^−1^, root production (7.33 and 5.00) became inhibited, and the number produced was significantly lower than that in the CK treatment (9.33).

As shown in Figure 3B, all three growth regulators had a significant promoting effect on the average maximum root length. The maximum root length of the cuttings in the IBA treatment group with a concentration of 1500 mg·L^−1^ was the largest (27.53 cm) among all the treatment groups; this value was significantly higher than that in the other treatment groups (across concentrations) and was 62.22% higher than that in the CK group. Second, the average maximum root length under the ABT-1 treatment fluctuated in the range of 17–23 cm; there was no significant difference between the different concentrations, but the length in response to the different concentrations were significantly higher than that in the CK treatment. The average maximum root length under the IAA treatment first increased and then decreased with increasing IAA concentration. However, there was no significant difference in average maximum root length between the concentrations or the CK.

As shown in Figure 3C, IBA at a concentration of 1000 mg·L^−1^ resulted in the greatest average maximum root diameter (2.18 mm), which was 67.89% thicker than that in the CK group (0.7 mm). The average maximum root diameter of the cuttings in the IBA treatment group with a concentration of 1500 mg·L^−1^ was slightly lower than that in the 1000 mg·L^−1^ IBA treatment group but was significantly higher than that in the 500 mg·L^−1^ and CK treatment groups. The average maximum root diameter under IAA treatment first increased and then decreased with increasing IAA concentration. The average maximum root diameter under the 500 mg·L^−1^ IAA treatment was the greatest (2.07 mm) and was 66.18% thicker than that under the CK. The average maximum root diameter under the ABT-1 treatment fluctuated in the range of 1.03–1.54 mm. Among the various ABT-1 concentrations tested, the average maximum root diameter under the treatment of 1500 mg·L^−1^ ABT-1 was the greatest, the value of which was significantly different from that under the CK. The average maximum root diameter in response to the other concentrations of ABT-1 was not significantly different from that of the CK group.

### 3.4. Changes in the Contents of Hormones in the Phloem of C. bignonioides Cuttings under Different Treatments

#### 3.4.1. Changes in the Content of IAA in the Phloem of *C. bignonioides* Cuttings

**Figure 4:**
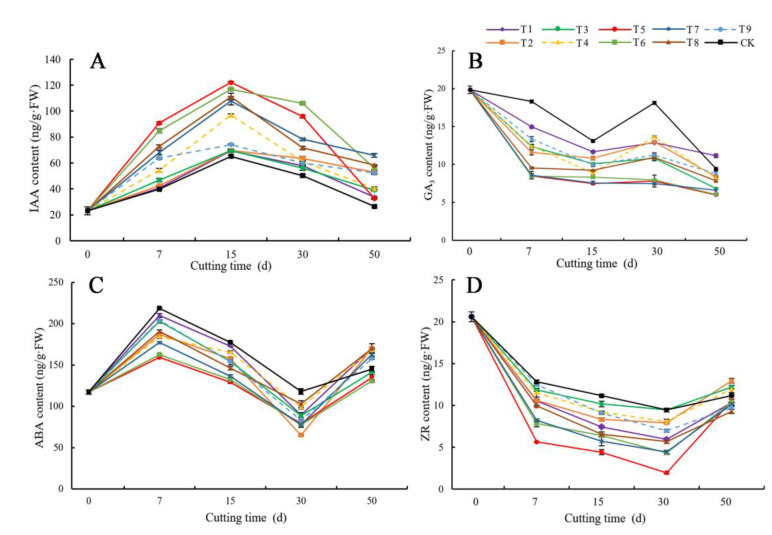
Changes in the content of IAA, GA_3_, ABA, and ZR in the phloem of *C. bignonioides* cuttings during rooting. (**A**) Changes in the content of IAA in the phloem of C. bignonioides cuttings during rooting, (**B**) Changes in the content of GA_3_ in the phloem of C. bignonioides cuttings during rooting, (**C**) Changes in the content ofABA in the phloem of C. bignonioides cuttings during rooting, (**D**) Changes in the content of ZR in the phloem of C. bignonioides cuttings during rooting, IAA: Indole-3-acetic acid; T1: IAA 500 mg·L^−1^ 1 h; T2: IAA 1000 mg·L^−1^ 1 h; T3: IAA 1500 mg·L^−1^ 1 h; T4: IBA 500 mg·L^−1^ 1 h; T5: IBA 1000 mg·L^−1^ 1 h; T6: IBA 1500 mg·L^−1^ 1 h; T7: ABT-1 500 mg·L^−1^ 1 h; T8: ABT-1 1000 mg·L^−1^ 1 h; T9: ABT-1 1500 mg·L^−1^ 1 h; CK: Control.

Figure 4A shows that the content of IAA in the phloem of *C. bignonioides* cuttings treated with growth regulators during the rooting process first increased and then decreased with time. Specifically, the IAA content increased on days 0–15 and decreased on days 15–50. Among them, treatment 5 corresponded to the largest increase. On day 15, the IAA content peaked at 122.10 ng·g FW, which was 5.26 times the initial content of the cuttings (23.22 ng·g FW). Moreover, the content was higher than that under the other treatments. The CK treatment yielded the smallest increase, and the value remained the lowest throughout rooting.

The rooting type and time points of the cuttings were analyzed. At the callus stage, the IAA content gradually increased. During the formation stage of adventitious roots, the content of IAA decreased. The analysis of the rooting effect of cuttings revealed obvious differences in IAA content among the treatments. Treatments in which the cuttings presented a high rooting rate also had a high IAA content. Among them, the cuttings under the CK had the lowest IAA content and exhibited the lowest root production. Taken together, these analysis results show that the higher the IAA content is, the better the rooting effect.

#### 3.4.2. Changes in GA_3_ Content in the Phloem of *C. bignonioides* Cuttings

As shown in Figure 4B, the GA_3_ content in the phloem of *C. bignonioides* cuttings during rooting showed a trend of “down–up–down” with time. On days 0–7, the GA_3_ content in all the treatments decreased sharply, with treatments 5, 6, and 7 showing the greatest decrease. On days 7–50, the GA_3_ contents in treatments 5, 6, and 7 were essentially stable, while those in the other treatments decreased by 50%. On the 15th day, the content of GA_3_ showed an increase–decrease trend again, among which the cuttings in the CK treatment presented the largest change. During the whole rooting process, the GA_3_ content in the CK treatment was always the highest. Among the various contents, the GA_3_ contents in treatments 5, 6, and 7 were maintained at lower levels.

The root morphology and root timing of the cuttings were analyzed. At the callus stage, the GA_3_ content decreased. However, the GA_3_ content increased during the root primordium and adventitious root generation stage. During the adventitious root elongation stage, the GA_3_ content decreased. According to the rooting analysis, the different growth regulators have obvious effects on the GA_3_ content and the range of variation within the cuttings. The lower the GA_3_ content, the stronger the rooting effect.

#### 3.4.3. Changes in Content of ABA in the Phloem of *C. bignonioides* Cuttings

Figure 4C shows that the ABA content of the phloem of *C. bignonioides* cuttings under each treatment during the rooting process showed a trend of “rising–falling–rising”. On days 0–7, the ABA content increased. Among the treatments, the ABA content in the CK increased the most, from the initial value of 117.19 ng·g FW to 218.51 ng·g FW, with an increase of 46.37%. On days 7–30, the ABA content decreased, but on days 30–50, the ABA content increased. On days 0–30, the ABA contents of all treatments were lower than those of the CK. Among the treatments, treatment 5 presented the lowest ABA content.

The rooting morphology and timing of the cuttings were analyzed. At the early callus stage, the ABA content increased. With the production of calli, root primordia, and adventitious roots, the ABA content decreased gradually. During adventitious root elongation, the ABA content began to increase again. Through the analysis of the effects of different growth regulators, concentrations, and treatment times on the cuttings, the lower the ABA content was, the stronger the rooting effect.

#### 3.4.4. Changes in the ZR Content of the Phloem of Cuttings of *C. bignonioides*

Figure 4D shows that the ZR content of the phloem of *C. bignonioides* cuttings under each treatment showed a trend of first decreasing and then increasing with rooting time. On days 0–30, the ZR content increased, but on days 30–50, the ZR content decreased. Among the treatment, treatment 5 presented the largest decrease in ZR content: at the initial cutting stage, the content was 20.59 ng·g^−1^ FW, and the lowest value was 1.93 ng·g^−1^ FW on the 30th day—a decrease of 90.63%. The decrease in ZR content in the CK was the smallest, from the initial value to 9.45 ng·g^−1^ FW on the 30th day—a decrease of 54.10%. Moreover, the ZR content decreased the sharpest in the CK treatment.

The rooting morphology and timing of the cuttings were analyzed. At the callus stage, the ZR content dropped sharply. During the root primordium generation and adventitious root formation stages, the ZR content continuously decreased, with a relatively small rate of decrease. During adventitious root elongation, the ZR content began to increase. Through the analysis of the effects of the different growth regulators, concentrations, and treatment times on the cuttings, the lower the ZR content was, the stronger the rooting effect.

#### 3.4.5. Changes in the IAA/ABA Value of the Phloem of *C. bignonioides* Cuttings

**Figure 5:**
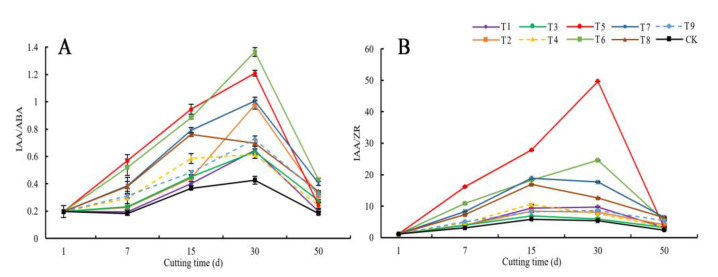
Changes in the IAA/ABA and IAA/ZR value of the phloem of *C. bignonioides* cuttings during rooting. (**A**) Changes in the IAA/ABA value of the phloem of C. bignonioides cuttings during rooting, (**B**) Changes in the IAA/ZR value of the phloem of C. bignonioides cuttings during rooting, IAA/ZR: Indole-3-acetic acid /Zeatin nucleoside; T1: IAA 500 mg·L^−1^ 1 h; T2: IAA 1000 mg·L^−1^ 1 h; T3: IAA 1500 mg·L^−1^ 1 h; T4: IBA 500 mg·L^−1^ 1 h; T5: IBA 1000 mg·L^−1^ 1 h; T6: IBA 1500 mg·L^−1^ 1 h; T7: ABT-1 500 mg·L^−1^ 1h; T8: ABT-1 1000 mg·L^−1^ 1 h; T9: ABT-1 1500 mg·L^−1^ 1 h; CK: Control.

Figure 5A shows that the IAA/ABA value of the phloem of the *C. bignonioides* cuttings under each treatment during the rooting process showed a trend of first increasing and then decreasing. On days 0–30, the IAA/ABA value increased, but on days 30–50, the IAA/ABA value decreased. (The IAA/ABA value for treatment 8 increased on days 0–15 and decreased on days 15–50.) The IAA/ABA values of treatment 5 and treatment 6 increased more than those of the other treatments did. The IAA/ABA value under the CK was always the lowest during rooting. The IAA/ABA values of treatment 5 and treatment 6 peaked on day 30 (1.21 and 1.36), which were 2.81 and 3.16 times the peak values in the CK (0.43), respectively.

The rooting morphology and timing of the cuttings were analyzed. The IAA/ABA values peaked from the initial period until the formation of adventitious roots. During the adventitious root elongation stage, the IAA/ABA values decreased sharply. Through the analysis of the effects of the different growth regulators, concentrations, and treatment times on the cuttings, the exogenous hormones promoted an increase in the IAA/ABA value. The higher the IAA/ABA value was, the stronger the rooting effect.

#### 3.4.6. Changes in the IAA/ZR Value of the Phloem of *C. bignonioides* Cuttings

Figure 5B shows that the IAA/ZR value of the phloem of the *C. bignonioides* cuttings under each treatment during the rooting process showed a trend of first increasing and then decreasing. On days 0–15, the IAA/ZR value increased, but on days 15–50, the IAA/ZR value decreased. (The IAA/ZR value for treatment 6 increased on days 0–30 and then decreased on days 30–50). Among them, treatment 5 presented the largest increase, from an initial value of 1.13 to a peak value of 49.65 on day 30, equal to an increase of 97.72%. The IAA/ZR value throughout the rooting process was the greatest in treatment 5. The IAA/ZR values of each treatment were higher than those of the CK.

The rooting morphology and timing of the cuttings were analyzed. The IAA/ZR value peaked from the initial period until the formation of adventitious roots. During the adventitious root elongation stage, the IAA/ZR value decreased. The lower the value was, the smaller the decrease. Through the analysis of the effects of the different growth regulators, concentrations, and treatment times on the cuttings, the exogenous hormones promoted an increase in the IAA/ZR value. The higher the IAA/ZR value was, the better the rooting effect.

## 4. Discussion

### 4.1. Effects of Growth Regulators on the Rooting Indexes of C. bignonioides Cuttings

According to related studies, rooting types of *C. bignonioides* softwood cuttings include the bark, callus, and mixed rooting types [16,36]. An abundance of research has shown that IBA treatment can significantly increase the rooting of cuttings [37,38]. However, some studies suggest that the rooting effect of cuttings is the greatest under treatment with ABT-1 [39]. Because of the ubiquitous endogenous IAA content plants, root development is always affected by changes in IAA contents. Numerous studies have shown that IAA can promote the rooting of cuttings *of Dalbergia serrata* [40], *Cinnamomum coninna* [41], and *Carnation* [42]. This study showed that three types of growth regulators can promote the formation of adventitious roots of *C. bignonioides* softwood cuttings. In particular, after IBA treatment, calli produced significantly earlier roots, and the rooting rate increased by 76.00% compared with that of the CK. In addition, the number of roots, root length, and rooting index were significantly higher than those in the CK group. Therefore, overall, the rooting effect of IBA treatment was the best, which is consistent with the research conclusions of most scholars of related studies [37,38].

### 4.2. Changes in the Contents of Endogenous Hormones Associated with Rooting

Plant endogenous hormones are important factors in the formation of adventitious roots on cuttings. Endogenous hormone levels are closely related to the rooting ability of cuttings [43]. IAA is the main hormone that promotes cuttings to develop adventitious roots [44]. According to most related studies [45,46] an increase in IAA content is beneficial to the formation and differentiation of root primordia. According to Taramino et al. high concentrations of IAA can increase the division of root meristem cells, while low concentrations of IAA can accelerate the differentiation of root elongation zone cells [47]. In rooting experiments on cuttings, such as *Chukrasia tabularis* and *Thuja occidentalis*, the content of IAA was found to be greatly increased during the critical period of adventitious root formation and decreased after adventitious root growth [28,48] In the present experiment, hormone treatments promoted IAA synthesis in cuttings, which induced adventitious roots. At this time, calli form in large numbers. Therefore, overall, an increase in IAA content can promote the differentiation of calli to form root primordia and induce rooting, which is consistent with the conclusion of Ma Zhenhua et al. [49] with respect to tetraploid *Robinia pseudoacacia*. Compared with the control group, the cuttings in the IAA-treated group rapidly accumulated, which was favorable for the induction and differentiation of root primordia. The main reason for the early rooting of cuttings may be the early and rapid increase in the IAA content. Therefore, IAA, IBA, and ABT-1 all promoted the IAA content in cuttings. In this experiment, IBA had the best effect and T5 had the best effect on IAA content.

Gibberellin GA_3_ mainly plays a role in promoting cell division and elongation and inhibits the formation of adventitious roots [50]. In a study on *Ipomoea fistulosa*, Nanda [51] et al. found that GA_3_ can significantly promote the rooting of cuttings and induce leaf bud germination. According to Li Yongxin et al. [33] high concentrations of GA_3_ inhibit the formation of adventitious roots. However, low levels of GA_3_ promote adventitious roots. In this experiment, the changes in the GA_3_ content in cuttings in the treatment group indicated that the formation and elongation of cutting adventitious roots was maintained at a lower level of GA_3_. The GA_3_ content remained at the lowest level in the IBA-treated group (T5), followed by the IBA-treated group (T6) and the ABT-1-treated group (T7). The GA_3_ content of the cuttings of the CK was maintained at a high concentration, and the rooting ability was poor, which also confirms the view that a high concentration of GA_3_ inhibits rooting [52]. Therefore, IAA, IBA, and ABT-1 treatment groups promoted the formation and elongation of cutting adventitious root by inhibiting GA_3_ content. In this experiment, IBA had the best effect.

ABA is considered a natural hormone in plants and has an inhibitory effect on the rooting of cuttings. The endogenous ABA content of cuttings of difficult-to-root tree species is much higher than that of easy-to-root species [53]. However, some studies [54] have suggested that high concentrations of ABA can promote the rooting of cuttings, the initiation of root primordia, and the formation and development of adventitious roots [55]. According to the results of this experiment, the ABA content in the cuttings showed a trend of increasing and decreasing at the early stage of rooting and then slightly rebounding, which may occur to increase the resistance of the cuttings and reduce the damage caused by stress. During the root primordium induction stage, the ABA content in the cuttings decreased sharply. After adventitious roots developed, the ABA content reached a minimum. Therefore, growth regulators affect the synthesis of ABA, and low levels of ABA are beneficial to rooting. The IBA treatment with the best rooting quality caused the ABA content of cuttings to be maintained at the lowest level, followed by ABT-1 treatment and IAA treatment. The ABA content of the cuttings in the CK treatment changed steadily throughout the rooting process and remained at a high level. In the absence of hormones, the change in the ABA content was small. Moreover, there was a negative correlation between the ABA content of each treatment group and the CK and the rooting ability. Therefore, IBA, IAA, and ABT-1 treatment groups inhibited the production of ABA in cuttings by regulating the changes in endogenous hormones in cuttings and promoted the formation of cutting adventitious roots. In this experiment, the IBA treatment group (T5 and T6) had the best effect.

ZR is the major transport form of cytokinin in woody plants; it plays a role in promoting cell division and differentiation, as well as in the formation of adventitious roots [56,57]. However, many researchers [57,58] have reported that the lower the ZR content, the better the rooting of plant cuttings, especially the induction of root primordia and the production of adventitious roots. The results of this study show that ZR content decreased sharply in the early callus period, which may promote callus induction. During the late callus stage, root primordium stage, and adventitious root generation stage, the ZR content decreased slowly, creating a low-ZR environment for root primordium induction and adventitious root production. After the adventitious root was generated, the ZR content gradually increased, and the elongation of the adventitious root was promoted, which was consistent with findings in *Soapberry* [59]. During the rooting process, the ZR content of cuttings in the treatment group was always lower than that in the control group. Exogenous hormone treatment reduced the ZR content of the cutting base to a certain extent and promoted the hydrolysis of starch and protein, as well as the generation of adventitious roots. Therefore, the IBA, IAA, and ABT-1 treatment groups inhibited the production of ZR in cuttings by regulating the endogenous hormone changes in cuttings and promoted the formation of cutting adventitious roots. In this experiment, the IBA treatment group (T5 and T6) had the best effect.

The rooting of cuttings is the result of the interaction of multiple plant hormones. Changes in the ratios of endogenous hormone contents can reflect the propensity for adventitious root formation [60]. According to studies by Guo Sujuan et al. [61] and Ao Hong et al. [56] high IAA/ABA and IAA/ZR values are beneficial to the rooting of cuttings, as well as the induction and differentiation of root primordia. According to the test results, the ratios of IAA/ABA and IAA/ZR in treatment group T5 cuttings with the best rooting effect all reached their peak on the 30th day after cutting. Therefore, the combined action of exogenous growth regulator influence and IAA, ABA and ZR promoted the formation of calli, the induction of root primordia, and the generation of adventitious roots. Compared with the CK group, the IAA/ABA and IAA/ZR ratios of cuttings in the IAA, IBA, and ABT-1 treatment groups peaked, which was favorable for the induction of root primordia. The main reason for the early rooting of cuttings was the early and rapid increase in the ratios of IAA/ABA and IAA/ZR. IAA, IBA, and ABT-1 treatment all promoted the increase in the IAA/ABA and IAA/ZR ratios. In this experiment, the IBA treatment group (T5 and T6) had the best effect. The results of this experiment support the research conclusions of Guo Sujuan et al. and Ao Hong et al.

## 5. Conclusions

In conclusion, the endogenous hormone content of cuttings changes in response to plant hormone treatments, thus affecting the rooting of the cuttings. During adventitious root induction, high levels of IAA and ZR and high IAA/ABA and IAA/ZR values favor root primordium induction in *C. bignonioides* softwood cuttings. The reduction in ABA and GA_3_ contents is beneficial to the rooting of cuttings. Therefore, IAA and ZR mainly promote *C. bignonioides* softwood cuttings, while ABA and GA_3_ play an inhibitory role. IBA treatment increased the IAA content during adventitious root induction and hastened the peak IAA content; however, IBA significantly decreased the ABA and GA_3_ contents. This is an important reason for the significant improvement in rooting ability after IBA treatment. The effects of different exogenous hormones on the dynamic physiological and biochemical processes and endogenous hormone contents and the specific mechanism through which endogenous hormones affect the production of adventitious roots of cuttings are complex and need to be further explored.

## Figures and Tables

**Table 1:** Orthogonal design of different growth regulator treatments. IAA: Indole-3-acetic acid; IBA: Indolebutyric acid; ABT-1: Rhizogenic powder-1; T1: IAA 500 mg·L^−1^ 1 h; T2: IAA 1000 mg·L^−1^ 1 h; T3: IAA 1500 mg·L^−1^ 1 h; T4: IBA 500 mg·L^−1^ 1 h; T5: IBA 1000 mg·L^−1^ 1 h; T6: IBA 1500 mg·L^−1^ 1 h; T7: ABT-1 500 mg·L^−1^ 1 h; T8: ABT-1 1000 mg·L^−1^ 1 h; T9: ABT-1 1500 mg·L^−1^ 1 h; CK: Control.

Treatment Number	A Growth Regulators	B Concentration (mg·L^−1^,)	C Treatment Time (h)
T1	IAA	500	1
T2	IAA	1000	1
T3	IAA	1500	1
T4	IBA	500	1
T5	IBA	1000	1
T6	IBA	1500	1
T7	ABT-1	500	1
T8	ABT-1	1000	1
T9	ABT-1	1500	1
CK	Water		

**Table 2:** Effects of growth regulators on the rooting rate and callus production rate of *C. bignonioides* softwood cuttings. IAA: Indole-3-acetic acid; IBA: Indolebutyric acid; ABT-1: Rhizogenic powder-1,CK: Control. The data in the table are the mean of 3 replications. The different lowercase letters (a, b, c, and d) after the data in the same column indicate significant differences at the *p* < 0.05 level, and the different capital letters (A, B, C, and D) indicate significant differences at the *p* < 0.01 level.

Growth Regulator	Rooting Rate/%	Callus Production Rate/%
IAA	32.89 ± 1.68 cC	8.22 ± 3.15 bAB
IBA	62.44 ± 2.14 aA	18.44 ± 3.67 aA
ABT-1	49.33 ± 1.76 bB	17.56 ± 5.35 aA
CK	8.00 ± 2.00 dD	2.00 ± 2.00 bB

**Table 3:** Effects of growth regulators on the root morphological indicators of *C. bignonioides* softwood cuttings. IAA: Indole-3-acetic acid; IBA: Indolebutyric acid; ABT-1: Rhizogenic powder-1; CK: Control. The data in the table are the mean of three replications. The different lowercase letters (a, b) after the data in the same column indicate significant differences (*p* < 0.05), and the different capital letters (A, B) indicate significant differences (*p* < 0.01).

Growth Regulator	Number of Roots/n	Average Maximum Root Length/cm	Average Maximum Root Diameter/mm
IAA	8.33 ± 2.91 bA	16.34 ± 7.38 abA	1.47 ± 0.28 aA
IBA	16.56 ± 3.42 aA	18.31 ± 0.88 aA	1.61 ± 0.30 aA
ABT-1	10.11 ± 3.79 bA	20.51 ± 0.62 aA	1.27 ± 0.19 aAB
CK	7.00 ± 3.00 bA	10.40 ± 1.13 bA	0.70 ± 0.10 bB

## Data Availability

All data generated or analyzed during this study are included in this published article.
